# P-604. Preliminary real-world Abrysvo vaccine effectiveness (VE) against Respiratory Syncytial Virus (RSV)-related lower respiratory tract disease (LRTD) hospitalizations and emergency department (ED) visits—Kaiser Permanente of Southern California (KPSC), November 2023-April 2024

**DOI:** 10.1093/ofid/ofae631.802

**Published:** 2025-01-29

**Authors:** Sara Y Tartof, Negar Aliabadi, Gabriella Goodwin, Jeff Slezak, Vennis Hong, Bradley Ackerson, Qing Liu, Sally Shaw, Sabrina Welsh, Julie Stern, Banshri Kapadia, Brigitte Spence, Joseph Lewnard, Gregory Davis, Michael Aragones, Michael Dutro, Erica Chilson, Elisa Gonzalez, Robin Hubler, Brandon Chia, Luis Jodar, Bradford D Gessner, Elizabeth Begier

**Affiliations:** Kaiser Permanente Southern California, Pasadena, CA; Pfizer, New York, New York; Kaiser Permanente, Southern California, Pasadena, California; Kaiser Permanente Southern California, Pasadena, CA; Kaiser Permanente, El Monte, California; Kaiser Permanente Southern California, Pasadena, CA; Pfizer Inc., Collegeville, Pennsylvania; Kaiser Permanente Southern California, Pasadena, CA; Pfizer, Inc, Collegeville, Pennsylvania; Kaiser Permanente Southern California, Pasadena, CA; Kaiser Permanente, Southern California, Pasadena, California; Kaiser Permanente Southern California, Pasadena, CA; University of California Berkeley, Berkeley, California; Kaiser Permanente, Southern California, Pasadena, California; Kaiser Permanente Southern California, Pasadena, CA; Pfizer, New York, New York; Pfizer, New York, New York; Pfizer, New York, New York; Pfizer Inc., Collegeville, Pennsylvania; Kaiser Permanente School of Medicine, South Pasadena, California; Pfizer Vaccines, Collegeville, Pennsylvania; Pfizer Biopharma Group, Collegeville, Pennsylvania; Pfizer Vaccines, Collegeville, Pennsylvania

## Abstract

**Background:**

While the pivotal randomized clinical trial documented the efficacy of the RSVpreF vaccine (Abrysvo) against RSV-related LRTD, the limited number of hospitalizations and ED visits hampered VE assessment for these clinical outcomes. We evaluated Abrysvo’s effectiveness against 1st occurrence of RSV-related inpatient or ED LRTD in KPSC, a large US healthcare system.

**Table 1.**
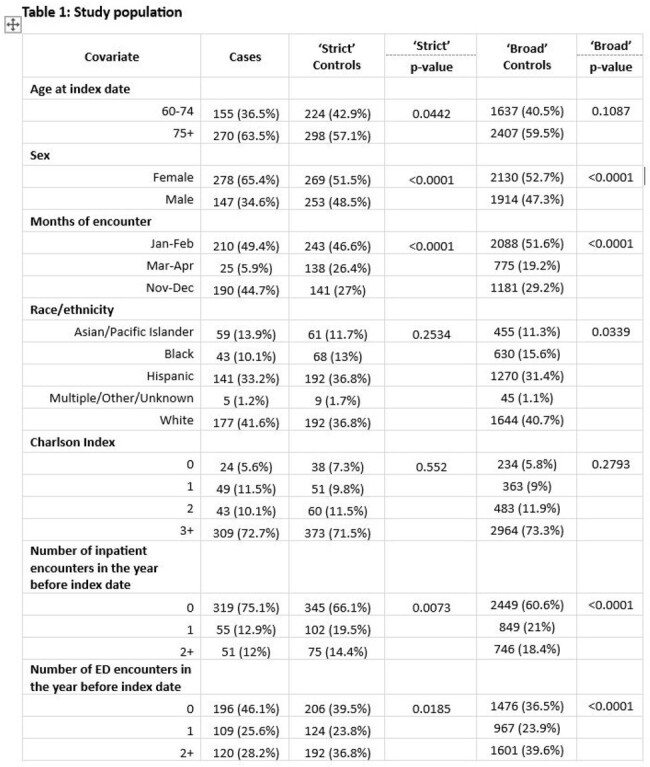
Characteristics of study population, KPSC

**Methods:**

In this test-negative case control study, we assessed VE among adults ≥60 years of age by analyzing KPSC members’ electronic health records and enhancing specimen testing from 11/24/2023-4/9/2024. Events from inpatient or ED settings meeting an ICD-10 code-based LRTD case definition with a respiratory specimen tested on GenMark RP2 expanded multiplex PCR assay were included. Cases were defined as RSV+ episodes. Analyses were conducted using two sets of pre-specified controls: 1) ‘Strict’: RSV- and positive for a non-vaccine preventable disease (VPD) etiology, and 2) ‘Broad’: all RSV-. The strict approach accounted for lower sensitivity of RSV testing among adults and potential bias associated with VPD controls. Exposure was defined as Abrysvo receipt ≥21 days before encounter. Adjusted odds ratios (OR) and 95% confidence intervals (CI) were estimated from a multivariable logistic regression model with adjustment for patient demographic and clinical characteristics. VE was calculated as 1−OR multiplied by 100%.

**Table 1.**
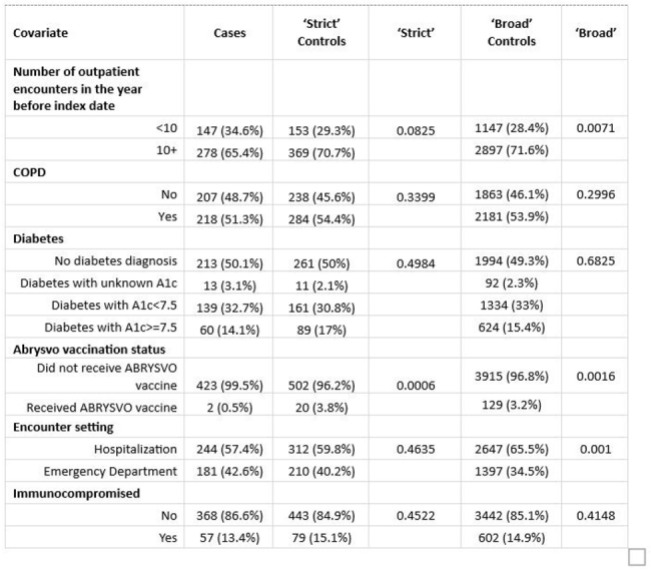
Characteristics of study population, KPSC, cont.

**Results:**

The overall study population included 4,469 KPSC members: >50% were ≥75 years, 93% had ≥1 comorbidities, and 15% immunocompromised. In the strict analysis, there were 425 cases and 522 controls who received Abrysvo a median of 59 days before illness onset; VE compared to no vaccine receipt was 84% (95% CI: 29–97). In the broad analysis, there were 425 cases and 4,044 controls who received Abrysvo a median of 53 days before illness onset; adjusted VE was 82% (95% CI: 28–96).

**Table 2.**
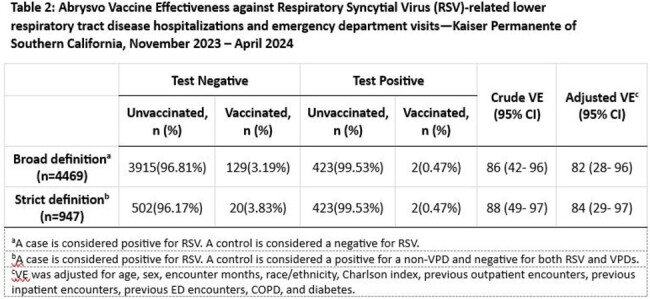
Abrysvo Vaccine Effectiveness against RSV LRTD hospitalizations and ED visits, KPSC

**Conclusion:**

This study demonstrated real world VE of Abrysvo during its first 5 months of use against severe LRTD in the hospital and ED settings among US adults ≥60 years of age. These results expand on efficacy results from clinical trial populations by including a substantial number of persons age 75+ years and with comorbidities and immunocompromising conditions.

**Disclosures:**

**Sara Y. Tartof, PhD MPH**, GSK: Grant/Research Support|Pfizer Inc: Grant/Research Support **Negar Aliabadi, MD, MS**, Pfizer Inc: employment|Pfizer Inc: Stocks/Bonds (Public Company) **Jeff Slezak, MS**, Dynavax Technologies: Grant/Research Support|Pfizer: Grant/Research Support **Vennis Hong, MPH**, Pfizer: Grant/Research Support **Bradley Ackerson, MD**, Dynavax: Grant/Research Support|GlaxoSmithKline: Grant/Research Support|Moderna: Grant/Research Support|Pfizer: Grant/Research Support **Qing Liu, M.S.**, Pfizer Inc.: Stocks/Bonds (Public Company) **Sally Shaw, DrPH, MPH**, Pfizer: Grant/Research Support **Sabrina Welsh, MPH**, Pfizer Inc: Stocks/Bonds (Public Company) **Julie Stern, MPH**, GlaxoSmithKline: Grant/Research Support|Pfizer: Grant/Research Support|Sanofi: Grant/Research Support **Michael Dutro, PharmD**, Pfizer: Stocks/Bonds (Public Company) **Erica Chilson, PharmD**, Pfizer Inc: Employee|Pfizer Inc: Stocks/Bonds (Public Company) **Elisa Gonzalez, MS**, Pfizer: Stocks/Bonds (Private Company) **Robin Hubler, MS**, Pfizer, Inc.: Employee of Pfizer Inc.|Pfizer, Inc.: Stocks/Bonds (Private Company) **Luis Jodar, PhD**, Pfizer Inc: Employment|Pfizer Inc: Stocks/Bonds (Public Company) **Bradford D. Gessner, M.D., M.P.H.**, Pfizer: Employee|Pfizer: Stocks/Bonds (Public Company) **Elizabeth Begier, MD, M.P.H.**, Pfizer Vaccines: Employee|Pfizer Vaccines: Stocks/Bonds (Private Company)

